# New Researches in the Departments of Physiology and Practical Medicine

**Published:** 1847-07

**Authors:** 


### ART. VIII.

*Neue Untersuchungen in den Gebieten der Physiologie und der practischen Heilkunde von* Dr. K. H. BAUMGAERTNER.—*Freiburg*, 1845.

*New Researches in the Departments of Physiology and Practical Medicine.* By Dr. K. H. BAUMGAERTNER.—*Friburg*, 1845. 8vo, pp. 402.

DR. BAUMGAERTNER, of Friburg, is one of those German physiologists to whose labours we are indebted for the natural history of cells. He claims precedence, indeed, in this field of discovery over Schleiden and Schwann. In the present work, he gives the further results of his researches in physiology and pathology, and which he wishes to be considered as a sequel to those undertaken by him in 1828 and 1829. The work is divisible into three parts: firstly, new researches into cell-life and development; secondly, sundry theories and hypotheses founded thereon; thirdly, empirical contributions to pathology. We shall notice analytically one or two of the more important matters contained in the first and third divisions of the work.

I. PHYSIOLOGICAL RESEARCHES. *The structure and development of muscular fibre.* Baumgärtner gives in detail the results of his researches into the embryonic development of muscular fibre, and into the microscopic anatomy, mode of reproduction, and relations to the tendons, nerves, and blood-vessels of the developed fibre of muscles, whether voluntary, involuntary, or mixed. We shall content ourselves with a general summary of the results.

Shortly after impregnation the ovum divides into globular halves; each of these divide into other two halves, and the latter into other two, and so on continuously, until at last the whole yelk is subdivided into small globules—the formative globules—which in the frog, are 0·003 inch in diameter.* A separation then takes place in different parts of the yelk between the globules, and they unite into groups, and from these various organs are formed. In a certain part of the yelk, namely, in that corresponding to the vertebræ, may be observed a number of globules united like strung pearls, some of which rows when united form a cord; these are the muscles in their first formation.

Every formative globule consists at first of conglomerated yelk-granules, with an amorphous matter; a change, however, soon commences in the centre of the mass, and a homogeneous colourless granule is formed. This granule gradually increases in size by appropriating to itself the surrounding yelk-granules, until at last it occupies the whole space of the formative globule. This cell does not consist of homogeneous matter as the primary granule, but is divided into minute parts, which have the appearance of small globules with minute granules, and of flattened corpuscles. From the shell-like covering (Schale) is formed the tissue, and from the minute globules arranged in a row, the fibres of the tissue. By the junction of the fibres of each cell with those of the next, muscular fibres are formed, and as each cell is open above and below a tube is formed by their junction.

The formative matter entering into each muscular fibre, or rather tube, gives origin to one or several series of new formative granules, which pass through the same series of changes as the original granules, and in this way new muscular tubes are formed one within another, like a nest of pill-boxes. Sometimes these inclosed tubes have the same axis as the parent tube, sometimes the axis is not the same: a difference of arrangement depending probably upon fixed principles.

In none of the different kinds of muscular tissue are the fibres merely longitudinal or placed in one direction only; they are all interlaced. In the voluntary muscles, the cross fibres are fine and perfectly round; in the inner layers and among the fine tubes, they cross the others, so as to give the tissues a tuft-like appearance; but in the outer layers and among the broader muscle-tubes, they present a net-like appearance. In both cases the interlaced web of muscle-tubes is so constituted, that there are two bundles of fibres, of which the one passes spirally to the right, the other to the left.

The so-called primitive muscular fibrils are not simply fibrils, but are made up of several finer, after an arrangement very similar to that by which the muscle-tubes are made up. On examining these carefully, there may be seen lucid points recurring at regular intervals which give the fibril a knotty appearance. These spots mark the points where the very

* This cleaving of the yelk is not yet proved to be general, although from the embryological observations of late years, it probably is so. We observe that Dr. Reid noticed it in the development of the ova of the nudibranchiate mollusca, as described in a paper published by him in the June number of the 'Annals and Magazine of Natural History' for 1846.

fine transverse fibrils that form the tuft-like tissue, cross the longitudinal fibrils; this shining point of junction appears, on a more minute examination, to present the appearance of a square projection.

The whole of the motor apparatus consists of regularly arranged muscle-tubes, between which cellular tissue is found, termed here *perimysium* by our author, but which appears to correspond to the *sarcolemma* of Bowman. The nervous and vascular trunks occupy these cellular interspaces. From the nerves smaller branches are given off, which loop round the bundles of muscle-tube, and return into the trunks from which they are given off, or else join to a neighbouring branch. No twigs can be traced into the muscle-tubes, and it seems certain that the nerves do not terminate in them, but only loop round them. The arterial capillaries are given off from the small arterial trunks in the perimysium, and looping round a bundle of muscle-tubes, end in the venous trunks which accompany the arterial. These details confirm in their more important points the researches of Bowman and others.

*The general structure of the skin.* Baumgärtner limited his researches into the structure of the skin to the relations of its principal tissues. These are three; 1. The epidermis, or cuticle. 2. The firm thick structure constituting the dermis. 3. A double network of vascular and nervous loops like those of the muscles, ending in the veins and the nervous trunks. These loops of nervous fibrils are easily traced in the mucous membrane also; as, for example, in that of a frog's throat. On the tongue, the nerves are distributed thus: a distinct twig passes to each papilla, and is there spread out; the course of the twigs is not easily followed, but Baumgärtner has no doubt that loops are formed which return to the main trunk.

*The structure of the brain.* Are the nerves and brain constituted alike? or are they distinct tissues? and if the latter, how are they connected? Baumgärtner examined the brain and spinal ganglia of the crayfish (das ganz kleiner Krebse); the latter to illustrate the former.

When a spinal ganglion is stripped of its investing membrane, and placed under the microscope, it presents the appearance of a flattened body made up of two striated halves, and the trunks of connected nerves. The middle of the ganglion is opaque, but it is apparent, that it has at that point no striated or fibrillar appearance, and is of a colour unlike that of the nerves. If pressure be applied, the two strands separate a little, and bundles of transverse fibres are observed, together with a mass in which transparent globules or vesicles are perceptible. On a closer investigation, this mass is found to consist of those globules which are connected with each other by a soft membranous tissue. The globules themselves when placed in water, are found to be cells containing one granule or several. The connecting material contains also cells of a smaller size. If the surface of the strands be examined at their entrance into the ganglion, numerous small oval cells are seen, and on closer observation broad membrane-like striæ are observed to inclose these cells and form a network with wide openings round the strands. If the latter be a little separated, this network is found to form a bridge of connexion between them. Besides these cells there are smaller, that are heaped up in masses here and there.

The spinal strands and lateral nerves may be readily traced into the ganglion; it is only in the centre that their course is obscure. Baumgärtner states the fact confidently, as observed by him that the brain consists of two tissues. 1. The proper cerebral substance made up of a layer of large and a layer of smaller cells, and which lie in a network. 2. The nervous fibrils entering into the ganglia which cross each other in various ways, and, forming loops, return to the stem from which they departed. Baumgärtner states that he has detected this structure in the brain of a chicken. Such of our readers as may attempt a verification of our author's statements, will do well to attend carefully to the cautions and directions given by him for the better observation of the structures he describes, since it appears that considerable management is requisite to get a sight of them. In particular, blood-vessels must not be mistaken for fibrils of nerves.

The cortical substance of the brain in animals of a higher organization appears made up of a soft membranous adherent mass and a network of vessels. Baumgärtner could detect only a few loop-like nervous fibrils running back into the medullary substance. The medullary substance appears to be made up of fibrils and a special network in which they lie. As a general summary of his researches, he observes that the nervous fibrils are distributed at their termination in the brain as at their termination in the muscles. They are partly collected in bundles and partly form loops, which, uniting, crossing, or decussating with others, return to the point of entrance and again join a main trunk.

*Structure of the nerves.* The researches of Ehrenberg and of his successors in histological inquiries have satisfactorily shown, that the nervous fibrils are tubular, and not simple threads. What do these tubes contain? What is the structure of the walls? The nerves of the crayfish when compared with those of man are like sacking compared with fine linen; they are therefore much more suitable for histological inquiry on these points. The fibrils of those proceeding from the cerebral and spinal ganglia have the following structures. They are flattened tubes made up of large oval cells, giving them a jointed appearance. If they be left in water for a while and their contents then squeezed out, the latter is found to be more or less fluid, and has the appearance of a granular mass, presenting here and there soft globules and fine cells, some of which may be also seen adherent to the inner surface of the tube. Many cells may be also seen on the margins of each tube, and it may be often observed that they are situate between the thin layers of its walls. The embryological development of the nerve-tubes is similar in all essential respects to that of the muscle-tubes. These researches of Baumgärtner appear to have been conducted without a knowledge of those of Foville.

*Physiological relation of the nerves to the skin, muscles, and brain.* The microscopic researches just related are a confirmation of the opinion generally received, that the nerves form loops at their terminal points, whether in the skin, muscles, mucous membranes, or brain. But Baumgärtner says, they are not lost in these structures. They are to be considered as long ellipses, the one segment of which is in the central axis, the other on the periphery. How do these loops act in the phenomena of motion and sensation? That the muscle-fibres must have an active share in the production of motion is obvious from the fact, that the loops do not universally pervade them, as well as from various circumstances connected with muscular action, and which has led to the general adoption of Haller's views of irritability, as an inherent property of muscular tissue. Baumgärtner differs from Haller and his disciples: he attributes muscular action to the opposite polar currents of the muscular fibrils developed in them by innervation. The nerve-fibrils are conductors; the muscular fibres are non-conductors. He thinks that the granules of the cells are the active bodies, and the surrounding tissue (perimysium) connects them into a whole and gives elasticity to the structure. When innervation occurs, the granules are attracted to each other and adhere, and thus shortening of the fibrils takes place. Of course when the muscle is relaxed, or innervation ceases, or the antagonistic muscle acts, a separation of the coherent granules takes place, and a consequent elongation of the muscle and a relaxation of the contraction. The tuft-like interlacing of the muscular tissue is, according to our author's views, admirably adapted to the mode of action here supposed. The theory has the merit of ingenuity, but we can hardly say that it is novel. It is analogous to that propounded some years since by Mr. Wharton Jones.

*Mode of sensation.* It is matter of observation, that the smallest possible point of the skin is sensitive; but there are not nervous fibrils reticulated over every point. It is therefore inferred that the direct or immediate agency of the sensitive nerve is not necessary to the communication of a sensation. It is certain, too, that the mere physical action of light on the retina is not all that is requisite to vision. The skin, Dr. Baumgärtner thinks, must be looked upon as consisting of conducting and non-conducting tissue; the former being the cutaneous fibrils, the latter the epidermis. In each sensory apparatus, a distinct chemical or physical process is developed by external agencies. The whole explanation is too long and too hypothetical for our pages, we will therefore substitute an observation on spectral illusions by the author.

"In consequence of the illness of one of my children, I had passed many sleepless nights and experienced much distress of mind. When the mournful scene was at last comiug to a close I threw myself on a couch, and found that a confused number of images arose in my brain as soon as I closed my eyes. The sick child had often directed its hand to my nose, and I now continually saw a hand stretched out to my face. I saw the body of a child, which at the abdomen ended in the body of a dog, and both portions contorted themselves in a peculiar manner. I further observed a long row of grinning spectres, a dog's head on a stump, a ship with broken masts, &c. I had my consciousness perfect, and meditated on these interesting phenomena. I could cause these spectral appearances to appear and disappear at pleasure. When the daylight fell on my eyes and the forms of surrounding things were obvious to me, the illusions disappeared; but the moment I shut my eyes and external visual influences were excluded, they reappeared." (p. 90.)

We are acquainted with a physiologist who had a similar, and perhaps better, opportunity of investigating the nature of cerebral sensorial changes, in consequence of an accidental laceration of the scalp. His consciousness was perfect also, as in Baumgärtner's case. He found that things in the room, which he did not regard attentively, but which only casually caught his eye, assumed various forms, but principally those of still or statue-like objects. The shadows cast on the wall from the watcher's candle assumed various grotesque and gigantic forms; the folds of the white coverlet appeared sometimes like beautiful marble statues, the trunks of which tapered away apparently *ad infinitum*—sometimes they were like laundresses at work, &c. The character of the spectres seemed to depend upon coincident excitor phenomena. Thus, a noise of a basin gave rise to a spectre of interminable rows of potters, all busily at work and hammering; then, the fire-irons making a slight noise, the scene changed, and there was row after row of blacksmiths, ending in a long perspective, each a repetition of the other, and all hammering away with the utmost noise and diligence. Sleep was in fact prevented by these spectral sounds and sights.

The following opinion of Baumgärtner is interesting, but we must for the present hesitate to admit its validity. The readers of this Journal will require no new grounds for an unbelief:

"I am of opinion that there is psychal activity at every point where the nerves are supplied with cerebral or ganglionic matter. I have convinced myself on this point with reference to the spinal cord of lower animals by numerous experiments. I have observed decapitated frogs, snails, eels, flies, wasps, crayfish, &c., to perform movements which were exactly in harmony with the position in which the animal was placed, which exhibited adaptation, and which could not be considered as mere reflex movements." (p. 91.)

*The opposite polarity of the tissues.* We pass over various points in nutrition to notice a theory propounded by our author, which is ingenious, to say the least of it, and which is capable of various applications. It has been already explained, that the muscular fibrillæ shorten only when acted upon by the motor nerve. If, however, a piece of muscle be separated from the body, together with a portion of its nerve, we find that, on repeated stimulation, the irritability is lost, and is only recovered after a period of rest. Since the nerve is entirely dissevered from the central axis, the recovered power of excitory movement cannot be derived from the latter; nor can it be in consequence of renewed *enhæmatosis*, because the circulation is equally cut off. Baumgärtner argues therefore, that the power must have been derived from the muscular tissue itself, and that in fact there is a sort of polar or ganglionic action between the two forms of tissue. As an analogous process in ganglionic action, he points out the increase in magnetic power of a magnet induced by an increase in the weight of the iron. Baumgärtner therefore concludes, that the terminations of nerves are not simply recipients and conductors, but are also active agents in innervation, and have a polar relation to the tissues in which they terminate. Another curious theory, or analogy, brought forward by our author in connexion with nutrition, is the opposite polarity of apparatuses, as of vision and the optic nervous centres. The reaction of cosmic agents within the living body also presents our author with a subject of curious inquiry, especially with reference to sleep. This, he observes, is dependent upon the law of alternate activity and repose, and not upon cosmic agents only. There is less stimulation during the night, and consequently the tissues rest, to recruit their power, or become "charged" again. During the state of activity in the day there is greater stimulation, and the accumulated power is "discharged."

*Organic motion.* The laws of organic or vital motion are sufficiently obscure to warrant our noticing Baumgärtner's theoretical views, especially as they are supported by observation. In fact, a knowledge of these laws is of the highest importance in practical medicine. It is not possible to understand fully the nature of congestion, inflammation, fever, &c., without being acquainted with the causes of organic movements. Baumgärtner has devoted considerable research to this subject, and has arrived at two principal results: 1. There is an organic attraction and repulsion, as well as an electric, a magnetic, or a galvanic. 2. In fully developed animals, having a complete nervous system, the movements are under its control. In the movements of the solids there is, as a result of innervation, an attraction and repulsion between the constituent parts, and, in the movements of fluids, the innervated containing tissue puts the fluid in motion.

The nature of the changes that occur in the ovum after impregnation, show that polarized currents are developed in it. The movements of the globules resemble the movements of particles on an electrified plate, or floating free in water. They unite to each other, then separate, and again unite, and so on continually, until material changes take place in them. It is to be noted too that the ovum divides into two distinct halves, or opposite poles, or centres, and each of them into two other halves or opposite centres, and so on as before described; polarization taking place continually and developing the formative globules. It is further to be observed, that there is a decussation of these organic currents, or lines of attraction and repulsion, just as Oerstadt has found in electro-magnetic currents.

The subsequent changes in the constituents of the ovum indicate the influence of a polarizing imponderable agent. After the yelk-globules have been worked up and the formative globules developed, an action takes place from the centre to the circumference of the latter—a repulsion—and a cell is formed. The linear arrangement of the cells in the formation of tissue is dependent (Baumgärtner argues) on organic currents, and the nutritive process is analogous to that of crystallization.

The above statements are illustrative of the *dualistic* theory now advocated in Germany by numerous physiologists; a theory intended to comprehend phenomena apparently the most remote and the most dissimilar, and which only requires the substantial support of demonstrative proof to render it universally prevalent. There is something extremely fascinating to the mind in a theory which includes in one category the phenomena of life in its minutest details, of molecular physics, of light, heat, electricity, and galvanism, and of the vast bodies that move through eternal infinite space, and submits them to the operation of the simple laws of terrestrial and celestial motion. The explanation of the motion of the blood by the dualistic hypothesis, as given by Baumgärtner, is ingenious; our space will not, however, allow us to enter upon it.

We have an interesting chapter on the development of blood-globules or cells: and another on the structure of the hydra viridis, both which we recommend to the notice of histologists: it appears from Baumgärtner's researches, that the hydra viridis is made up of three layers of cells, (cells not united to form a tissue, but each an independent cell;) the inner layer is the stomach, the outer is the sensitive layer, the middle the motor.

There are no traces of nerves or of blood-vessels; the cells draw their own nutriment from the food taken into the stomach, and act also as conductors and motor agents.

*A contribution to morphology.* The next chapter is so curious a morphological fragment that we are compelled to notice it. Our author applies the dualistic theory to the development of the skeleton, and propounds the doctrine, that the latter is neither more nor less than a double ossific cell. He enters into details to show that at first an animal consists of one cell only, and that this divides into two cells (blase), an anterior and a posterior, inclosed in a common membrane. Each of these divide into two lateral halves, and into an upper and a lower half, and then into further subdivisions: on one side, the brain and spinal cord being developed with the posterior spinal column and the connected muscles, on the other, the anterior spinal column, the lungs, and the abdominal viscera. Lastly, from each of the two primary body-cells (körper blasen) four projections arise—those springing from the posterior cell being developed into the upper and lower jaw, those from the anterior corresponding to the four extremities. We need not prosecute these curious views further; at present they are merely curious.

*Cells.* The next chapter is on globules and cells. In this, Baumgärtner claims the discoveries usually attributed to Schwann and Schleiden. He refers to his elements of physiology and general pathology (published in 1835) for views, facts, and researches, like those brought forward by Schwann in 1839, when he propounded the theory of cells. He also claims for his views more completeness than is exhibited by those of the cell theorists, inasmuch as the latter bring forward no plan or theory which connects the multitudinous cells into an individual mass, whereas his dualistic theory attains this. We must leave the question of priority to be settled in Germany, simply observing, that Baumgärtner claims priority of discovery over Schwann by four years. In the present and succeeding chapter, is a review of the whole doctrine of cells founded on personal researches. We propose ere long, to give our readers a review of the cell-theory, which shall be supplementary to that in which we some years ago introduced it to our readers, and which shall include the dualistic doctrines propounded in this chapter by Baumgärtner, should future inquiries confirm his.

A chapter on "Animal chemistry" is devoted to an application of the dualistic theory (or theory of animal polarity) to the processes of nutrition and secretion. The elective affinities displayed by various tissues for the different constituents of the blood, are explained by our author with the aid of his theory. His views are, however, too hypothetical to be presented to our readers in detail: we therefore simply indicate where they are to be found.

II. Pathological Researches. *Nature of parasitoid formations.* Tubercles first appear in small, gray, semitransparent points. If such a small mass be removed and examined, it is found to be divisible into smaller masses. If examined by the microscope, these are seen to consist of small granular bodies and globules, the latter being contained in a granular cell, which adheres to the next adjoining cell. Baumgärtner thinks there is no doubt, that the formation of tubercle commences with the formation of simple granular bodies, that these cohere, and a globule is formed, and after that a granular cell, the process being in fact analogous to that exhibited in the development of the ovum.

The caseous matter of tubercles consists of small granular bodies which are not widely dissimilar from the formative globules (previously described) of the frog's ovum, such as they appear immediately after the formation of granules. If the caseous matter be in a more advanced stage of softening, it is found to consist almost altogether of globules, or cells containing a granule, their parietes being also somewhat granular. In proportion as the tubercular matter approaches the stage of suppuration, the granular bodies disappear, and the pus-globules (having a cell contained in a cell) become more numerous and interspersed with transparent molecules.

From these and similar results of microscopical investigation, Baumgärtner is led to think, that tubercle is a species of false membrane, and that their formative process is analogous, in all respects, to the formative process observed in the development of the ovum. The granular bodies are analogous to those of the yelk, which they resemble; out of them, as out of the latter, formative globules are developed, and then these latter change into cells. Here, however, the analogy ceases. The cells of the healthy process unite together to form the different tissues; the pus-cells remain in this low stage of development.

We pass over a long discussion on the pathology of heterologous formations, and also another on fever. In the latter, Baumgärtner applies his doctrines of dualism to the elucidation of febrile phenomena, and advances many ingenious hypotheses, which unfortunately are hypotheses only. He moots, for example, the idea already advanced by Schultz, that the periods of febrile diseases may depend upon the periods of development and decline of morbid cell products circulating with the blood. But then there is no proof advanced; it is a mere supposition, and so on with others. Our author's chapter on inflammation and its products is, in all respects, similar to that on fever. The dualistic cell-theory is applied to its pathology, but rather by hypothetical ratiocination than demonstration.

A chapter on Therapeutics contains some clear general applications of the previous theories to the cure of disease. One of these is, the destruction of parasitic animals by inunction. No plant, or animal, nor even a solitary cell can exist without an indirect or direct communication with the atmosphere. An oil enema destroys ascarides; and illination with olive oil will cure the itch: it seems not improbable that the method of cure in other cutaneous diseases that are removed by inunction is similar. Temperature has also an important effect. Aphthous products do not well bear a diminished temperature; consequently, cold gargles will be beneficial. Cold or warm remedies may be also applied locally. Poisonous applications have been extensively used in cutaneous and aphthous affections, as borax, alum, mineral oxides, salts, and acids, sulphur, iodine, phosphorus, &c. It is not improbable that arsenic, administered internally in cutaneous affections, acts as a poison on the morbid products, and might be used very effectually as an external application. Of course it would require the greatest care in its management.

A chapter entitled: "Final observations on the dualistic theory of life," is a singular composition. It extends the theory into cosmogonic metaphysics, if we may be allowed to connect two such words. It considers the theory of creation, of God's existence, the metaphysical notions of Descartes, Spinoza, Hegel, Schelling, &c.; the cosmogonical ideas of Laplace, Humboldt, &c. (applying dualism to an explanation of the theory of planetary formation, with which the British public has been lately made acquainted by the author of 'Vestiges of the Natural History of Creation;') and links the smallest particles of organized matter with the mighty masses of infinite space.

III. Clinical Contributions. *Typhus fever.* It was with some interest that we entered upon the perusal of these clinical contributions, because we were curious to ascertain, whether the study of transcendental physiology had impaired the practical tact and therapeutic skill of the writer. We are bound to say that no such noxious influence appears to have been excited on his mental powers or professional ability. He descends very gracefully from the sublime to the common-place. Ulcerative or abdominal typhus appears at Freiburg, ordinarily with an adynamic type. There is a febrile condition with little vascular excitement, a depression of the system, the typhoid countenance and tongue, and small pimples, or stigmata (frieselblasen) on the breast,—a very common symptom in one form of pestilential fever in the middle ages—the febris stigmatica. There was also diarrhea, bronchitis, vertigo, and slight delirium. Active cerebral excitement rarely occurred, and ecchymoses, petechiæ, or hemorrhages, in few cases only. The gastro-enteric, or mucous form of typhus, was not of frequent occurrence. In some cases the bronchitis masked the typhus, and the symptoms were not unlike acute phthisis; in others a complication with pneumonia led the most acute practitioner into mistaking typhus for the latter disease. The expression of countenance, however, led to a correct diagnosis.

*Treatment of typhus at Freiburg.* Baumgärtner, after trial of different methods, relies upon the expectant method as the best. At the commencement he ventures on an emetic, and he is inclined to think that it has occasionally "cut short" the disease, but on this point he speaks doubtfully, as the cases thus treated successfully might not have been true examples of the disease. He says, "I have treated many cases of typhus without administering any potent remedy throughout the whole course of the disease. Small doses of ipecacuan, or liquor of acetate of ammonia have been given, and these because patients are apt to lose confidence, and be dispirited unless something medicinal be done. The cases in which he adopts the expectant method are those in which the excretions are not suppressed, in particular those in which there are urinary deposits. In the first stage of cases complicated with pneumonia and bronchitis, he ventures on large doses of calomel, and occasionally a general bleeding; but in the second stage the calomel has been decidedly hurtful. In cases of extreme prostration, he administers wine freely, and in the rare examples in which the pulse is intermittent, and cramps take place, he places the patient in a warm bath, uses gentle cold affusion on the head, and administers musk.

*Treatment of angina tonsillaris* (rachencroup). Baumgärtner speaks of cauterization with lunar caustic as the most effectual of all means in angina tonsillaris, when false membranes form. He has never lost a patient from this disease since he adopted this method of cure. Incision of the inflamed tonsil is another effectual means of relief.

*Colourless hemorrhage.* Under this term Baumgärtner wishes to describe cases of flux in which the blood-globules are retained, and the colourless serum, plasma, or lymph, only passes out of the circulation. It is to be seen on a small scale as a sequel to hemorrhage from a small wound. It occurs principally after spontaneous hemorrhages, as from the uterus. In one example of this kind which Baumgärtner has described, the colourless hemorrhage was so profuse after the ordinary flooding, that the fluid soaked through a hair and a straw mattress, and flowed upon the floor; and he is of opinion, that similar cases (the discharge being in a more moderate degree) are often considered as, and mistaken for leucorrhœa. He would also classify under this head, the serous fluxes which occur from the bowels after diarrhea, or dysentery, and thinks that they are dependent upon a structural change in the mucous epithelium and subjacent parts, such that the serous contents of the capillaries escape. This serous or lymph-like discharge from the bowels has occasionally come under our own notice as a sequel of hemorrhage, and we believe has retarded the recovery very materially. We have found astringent enemata useful.

Effusion into the serous cavities is, in many cases, to be considered as an example of colourless hemorrhage. To this class, Baumgärtner thinks those cases are to be referred, in which the effusion is rapid, and unaccompanied by inflammatory symptoms, and particularly when blood-globules are here and there observed floating in the fluid.

*Prison scrofula.* Baumgärtner refers to scrofula as being one of the opprobria medicorum. The supplies of scrofulous cases are derived principally from orphan asyla, infirmaries, and particularly houses of detention. He has carefully examined the hygienic condition of the prisons in his neighbourhood, and he indicates the causes of the excessive prevalence of scrofula amongst them. Firstly, it appears that the prisoners are principally employed in the dusty and sedentary employment of weaving; secondly, there is a neglect of proper ventilation; thirdly, a want of proper food. Commonly, the prisoners have a *small* portion of animal food once only in eight days, but Baumgärtner knows prisons in which it is given out only once a fortnight!

The remaining articles in the volume are on the various forms of gastrodynia; a case of poisoning by strychnia, belladonna, and prussic acid (an apothecary taking his own drugs); an interesting case of aneurism of the root of the aorta; and a short essay on the prevention of the plague. The principle he recommends for accomplishing the latter is a modification of the morbid poison, by first passing it through the bodies of brutes, and then inoculating man, as in vaccination, the vaccinia being considered a similar modification of variola—an old suggestion. Numerous plates, illustrative of histological anatomy, are appended.

